# Conjugated linoleic acid content of human plasma

**DOI:** 10.1186/1476-511X-7-34

**Published:** 2008-09-30

**Authors:** Spiros N Zlatanos, Kostas Laskaridis, Angelos Sagredos

**Affiliations:** 1 Laboratory of Organic Chemistry, Chemical Engineering Dept., Aristotle University of Thessaloniki, GR-541 24, Thessaloniki, Greece

## Abstract

Conjugated linoleic acid (CLA), a naturally occurring anticarcinogen found in dairy products, is an intermediary product of ruminal biohydrogenation of polyunsaturated fatty acids. Few data exist on the CLA content of the human blood plasma. The determination of a "normal" content could help in estimating if a person consumes satisfactory amounts of CLA with the diet and thus takes advantage of its potential beneficial effects on health. The purpose of this study was to compare the plasma CLA content of individuals not consuming dairy products (group 1, n = 12), individuals consuming normal amounts of dairy products (group 2, n = 77) and individuals consuming CLA supplement (group 3, n = 12). The only CLA isomer that presented higher percentage than the detection limit (0.03% of total fatty acids) was rumenic acid (cis9, trans11-octadecadienoic acid). An interesting finding is that compared to the other two groups, group 3 members show the highest average plasma content in rumenic acid, i.e. 0.20% of total fatty acids. The present study could be characterized as the first step in the direction of establishing a normal CLA content of human plasma. Based on these results, it could be suggested that the lower limit of the plasma CLA content is approximately 0.1% of total fatty acids.

## Background

Conjugated linoleic acid (CLA) is a term that refers to a group of linoleic acid isomers with conjugated double bonds. In the past two decades there has been an increasing interest in the biological effects of CLA, since Grimm and Pariza [1] reported that dienes in fried ground beef protect against chemically induced cancer. The double bonds of the various CLA isomers could be in positions 7, 9; 8, 10; 9, 11; 10,12; or 11, 13 with *cis *or *trans *configuration, but the most abundant isomer in nature is *cis*-9, *trans*-11 [2,3]. This isomer is called rumenic acid and is formed by the anaerobic bacteria *Butyrivibrio fibrisolvens *in the rumen [4,5].

The richest food sources of CLA are first the dairy products, especially cheese and then ruminant meat [6,7]. CLA isomers can also be produced by heating linoleic acid in the presence of alkaline solutions. The isomerisation of linoleic acid to conjugated dienes was initially reported by von Mikusch [8].

Several animal experiments relate the consumption of CLA to the inhibition of tumorgenesis [9-11] and atherogenesis [12-14], the normalizing of glucose tolerance [15], and the reduction of body fat [16-18]. In several nutritional studies the effect of CLA supplementation on human body composition has been studied. However, the results of these studies showed that CLA had less effect on body fat than it was concluded from *in vivo *studies. A recent meta-analysis [19] has concluded that a dose of 3.2 g CLA/d produced a modest reduction in fat mass in humans.

It is known that rumenic acid is normally found in human plasma as part of triglycerides, phospholipids and cholesterol esters [20]. However, few data exist concerning the normal concentrations of CLA in human plasma. This kind of information could help to estimate if a person takes satisfactory amounts of CLA with the diet or if there is a need to increase them by supplementation, given its potential effects on health-related parameters.

Mougios *et al *[21] showed that diet supplementation with small doses of CLA in encapsulated form for 4–8 weeks, may increase the CLA content in human serum. There is no indication if this increase is within the normal levels of a person consuming dairy. Therefore, in the present study, an effort was made to set a "limit" between low and high plasma CLA content. In this direction the plasma fatty acid composition was determined in 101 volunteers divided into three groups: a group not consuming any dairy products, a group consuming normal amounts of dairy products and a group consuming CLA supplements for several months.

## Materials and methods

### Participants

Group 1 is comprised of 6 women and 6 men.

Group 2 is comprised of 37 women and 40 men.

Group 3 is comprised of 6 women and 6 men.

Blood samples used in the current study were collected from German volunteers. The age of the volunteers ranged from 18 to 65 years. Individuals were not affected by gastrointenstinal or liver diseases, which could influence fatty acid metabolism. All participants were interviewed about their dietary habits and especially about the consumption of milk, cheese and other dairy products. Another 12 volunteers were invited to participate in the study, as they were daily consuming 4 × 500 mg soft gelatin capsules of CLA (CLA 70-Trofocell™, Trofocell™ Ltd, Hamburg, Germany) for a period of 6 months. This Trofocell supplement contains about 35% of cis9, trans11-octadecadienoic acid (rumenic acid) and an equal amount of *trans*-10, *cis*-12-octadecadienoic acid.

### Sample preparation

8–10 mL of venous blood samples were collected in heparin tubes. After centrifugation at 4000 rpm the supernatant blood plasma was separated with a pipette. The blood samples were collected in the morning in the fasted state after a 12 h restriction for meal and drinks.

### Preparation and analysis of fatty acid methylesters

To 1 g of plasma, 5 mL n-KOH solution in 90% watery methanol and 0,20 mg heptadecanoic acid methyl ester internal standard in 1 mL hexane were added and the solution was saponified at 4°C overnight. 15 mL of 10% BF_3 _solution in methanol (BF3 methanolate, Merck) were added and the solution was heated at 60°C for 15 min. Then, it was extracted with 25 mL of hexane. The hexane phase was evaporated in a rotary evaporator until dryness. The residue was dissolved in 0.3 mL of hexane and analyzed with gas chromatography, according to Sagredos & von Leitner [22].

In brief:

### Gas chromatography – Conditions

*Apparatus*: HEWLETT-PACKARD Auto system; *Column*: Sil 88, 50 m, 0.25 ID, 0,25 μm Film thickness; *Detector*: FID (300°C); *Injection*: 3 μL split 1 to 50 (250°C); *Initial pressure*: 22 psi He; *Temperature program*: 60°C for 5 min isotherm, 5°C/min to 180°C, 16 min isotherm, 5°C/min to 220°C, 15 min isotherm.

The fatty acid methyl esters were identified by comparing their retention times to those of standards. The CLA methyl esters standards were obtained from Cayman (Ann Arbor, Ml), while the other standards were obtained from Sigma-Aldrich. The weight of CLA and that of the other fatty acids were obtained from the chromatographs and were transformed to weight percentages (%) of total fatty acids in comparison to internal standard and by utilizing response factors. The quantification limit was 0.03% of total fatty acids. Conventional statistics routines (i.e. Excel spreadsheets) were employed for the statistical analysis.

## Results and discussion

The plasma fatty acid composition of the three participating groups examined.

### Group 1

It comprises 12 individuals, who did not consume any dairy products.

### Group 2

In this group 77 volunteers were participated, who consumed normal amounts of dairy products. It means a consumption of about 300–400 g cheese/week. Cheese is the CLA richest dairy product and the main nutriment for a CLA intake. The weekly intake of milk or other dairy products has not been considered because of their relative low concentrations of CLA [23].

### Group 3

It comprises 12 participants, who daily, after lunch or dinner, were supplied with 4 × 500 mg CLA Capsules of a total amount of 1.4 g CLA (= 0.7 g rumenic acid and 0.7 g t10, c12-CLA).

**Figure 1:**
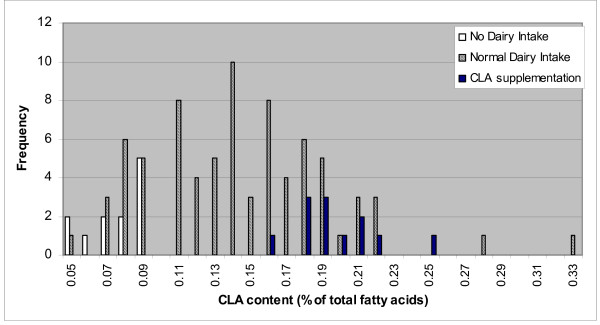
Distribution of the CLA values among the three groups of individuals.

The analytical results are summarized in Tables 1, 2 and 3 respectively. In all samples analyzed only the *cis*-9, *trans*-11 isomer of CLA was found in amounts higher than the detection limit. The rumenic acid content of group 1 ranged from 0.05 to 0.09% and averaged 0.08% of total fatty acids. The rumenic acid content of group 2 ranged from 0.05 to 0.33% and averaged 0.14% of total fatty acids, significantly higher than the rumenic acid content of group 1 samples. Finally, the rumenic acid content of group 3 ranged from 0.16 to 0.25% with an average of 0.20% of total fatty acids, significantly higher than the rumenic acid content of the two others groups. The distribution of the rumenic acid values among the three groups of individuals is presented in Figure 1. An interesting finding is that only 15 of the 77 samples of the second group present rumenic acid content comparable to the first group. Group 1 exhibits an average plasma content in rumenic acid of 0.08% of total fatty acids, significantly lower than of group 2, i.e. 0.14% of total fatty acids. It is noteworthy, that group 3 exhibits the highest average plasma content in rumenic acid, i.e. 0.20% of total fatty acids.

**Table 1:** Blood plasma fatty acid composition (% of total fatty acids) of individuals not consuming dairy products (n = 12) (group 1)

**Fatty acid**	**Occurrence of value (>0.03%)**	**Median**	**Average**	**Standard Deviation**	**Minimum**	**Maximum**
C 12:0	12	0.12	0.13	0.09	0.05	0.38
C 14:0	12	1.05	1.11	0.56	0.49	2.60
C 14:1ω5	12	0.08	0.09	0.05	0.03	0.21
C 15:0	12	0.22	0.26	0.09	0.17	0.50
C 16:0	12	23.3	23.39	2.70	18.5	27.70
C 16:1ω7 t	12	0.20	0.21	0.07	0.08	0.40
C 16:1ω9 c	12	0.31	0.32	0.09	0.21	0.48
C 16:1ω7 c	12	2.10	1.94	0.79	0.87	3.30
C 17:1ω7	7	0.14	0.13	0.04	0.07	0.17
C 18:0	12	8.40	8.13	1.18	5.80	10.2
C 18:1ω9 t	12	0.32	0.30	0.06	0.16	0.37
C 18:1ω7 t	9	0.31	0.32	0.12	0.13	0.58
C 18:1ω9 c	12	16.85	18.23	3.00	15.00	24.50
C 18:1ω7 c	12	1.40	1.50	0.32	1.10	2.20
C 18:1ω6 c	12	0.13	0.14	0.04	0.08	0.23
C 18:2ω6 tt	10	0.08	0.08	0.04	0.03	0.17
C 18:2ω6 ct	12	0.17	0.19	0.04	0.14	0.28
C 18:2ω6 tc	12	0.14	0.14	0.04	0.09	0.23
C 18:2ω6 cc	12	25.75	25.23	4.96	16.9	32.1
C 20:0	9	0.06	0.07	0.02	0.04	0.12
C 18:3ω6	12	0.39	0.34	0.12	0.12	0.50
C 20:1ω9	12	0.13	0.17	0.09	0.09	0.42
C 18:3ω3	12	0.51	0.54	0.21	0.33	1.00
c9, t11-CLA	12	0.08	0.08	0.01	0.05	0.09
t10, c12-CLA		<0.03	<0.03			
C 18:4ω3	3	0.24	0.24	0.02	0.21	0.26
C 20:2ω6	12	0.23	0.22	0.02	0.17	0.25
C 22:0	11	0.15	0.16	0.05	0.08	0.25
C 20:3ω6	12	1.35	1.39	0.23	0.96	1.80
C 20:4ω6	12	7.20	6.83	1.57	4.00	9.20
C 20:5ω3 (EPA)	12	0.81	0.88	0.35	0.17	1.50
C 22:4ω6	12	0.22	0.22	0.05	0.11	0.33
C 22:5ω3 (DPA)	12	0.65	0.67	0.15	0.42	1.00
C 22:6ω3 (DHA)	12	2.40	2.55	0.85	1.20	4.30
Total fatty acids ( μg/g)	12	2464	2581	669	1483	3948

**Table 2:** Blood plasma fatty acid composition (% of total fatty acids) of individuals consuming normal amounts of dairy products (n = 77) (group 2)

**Fatty acid**	**Occurrence of value (>0.03%)**	**Median**	**Average**	**Standard Deviation**	**Minimum**	**Maximum**
C 12:0	76	0.19	0.22	0.13	0.07	0.74
C 14:0	77	1.40	1.50	0.64	0.49	3.30
C 14:1ω5	76	0.10	0.12	0.07	0.03	0.35
C 15:0	77	0.33	0.33	0.11	0.13	0.77
C 16:0	77	24.40	24.34	2.63	17.00	29.90
C 16:1ω7 t	77	0.23	0.23	0.08	0.05	0.43
C 16:1ω9 c	76	0.36	0.36	0.11	0.07	0.88
C 16:1ω7 c	77	2.00	2.15	0.89	0.36	4.70
C 17:1ω7	57	0.17	0.16	0.06	0.03	0.30
C 18:0	77	7.60	7.78	1.11	5.20	11.9
C 18:1ω9 t	70	0.355	0.37	0.17	0.13	1.30
C 18:1ω7 t	58	0.32	0.34	0.13	0.06	0.64
C 18:1ω9 c	77	19.3	19.44	3.10	11.6	28.2
C 18:1ω7 c	77	1.50	1.51	0.34	0.92	2.80
C 18:1ω6 c	59	0.10	0.11	0.04	0.03	0.18
C 18:2ω6 tt	72	0.08	0.09	0.05	0.03	0.23
C 18:2ω6 ct	77	0.19	0.20	0.05	0.12	0.33
C 18:2ω6 tc	77	0.14	0.15	0.04	0.08	0.28
C 18:2ω6 cc	77	24.20	24.81	5.01	14.20	37.40
C 20:0	74	0.06	0.08	0.05	0.02	0.24
C 18:3ω6	77	0.29	0.30	0.11	0.08	0.83
C 20:1ω9	77	0.15	0.15	0.05	0.06	0.30
C 18:3ω3	77	0.50	0.59	0.41	0.25	3.30
c9, t11-CLA	77	0.14	0.14	0.05	0.05	0.33
t10, c12-CLA		<0.03	<0.03			
C 18:4ω3	15	0.12	0.14	0.07	0.06	0.35
C 20:2ω6	77	0.22	0.22	0.05	0.13	0.40
C 22:0	74	0.15	0.16	0.06	0.04	0.34
C 20:3ω6	77	1.40	1.39	0.34	0.57	2.30
C 22:1ω11	2	0.035	0.04	0.01	0.03	0.04
C 20:4ω6	77	5.30	5.41	1.43	2.50	9.10
C 20:5ω3	77	0.64	0.75	0.44	0.19	2.50
C 22:4ω6	76	0.19	0.19	0.06	0.04	0.41
C 22:5ω3	77	0.52	0.56	0.16	0.34	1.10
C 22:6ω3	77	1.80	1.91	0.77	0.70	4.30
Total fatty acids ( μg/g)	77	2553	2834	890	1438	6093

**Table 3:** Blood plasma fatty acid composition (% of total fatty acids) of individuals consuming CLA supplements*) (n = 12) (group 3)

**Fatty acid**	**Occurrence of value (>0.03%)**	**Median**	**Average**	**Standard Deviation**	**Minimum**	**Maximum**
C 12:0	7	0.20	0.25	0.10	0.14	0.39
C 14:0	12	1.80	1.72	0.73	0.55	2.70
C 14:1ω5	4	0.20	0.15	0.09	0.04	0.27
C 15:0	12	0.39	0.42	0.20	0.18	0.77
C 16:0	12	25.40	25.53	1.72	22.50	28.80
C 16:1ω7 t	12	0.20	0.22	0.12	0.05	0.38
C 16:1ω9 c	12	0.42	0.42	0.23	0.07	0.88
C 16:1ω7 c	12	2.60	2.57	1.13	4.70	0.66
C 17:1ω7	11	0.19	0.18	0.08	0.07	0.27
C 18:0	12	6.70	6.01	1.08	5.90	9.20
C 18:1ω9 t	12	0.45	0.43	0.16	0.17	0.69
C 18:1ω7 t	6	0.17	0.26	0.16	0.13	0.49
C 18:1ω9 c	12	20.30	19.99	2.29	15.50	23.50
C 18:1ω7 c	12	1.50	1.61	0.40	1.10	2.20
C 18:1ω6 c	8	0.11	0.12	0.03	0.08	0.16
C 18:1ω6 tt	12	0.11	0.11	0.06	0.04	0.21
C 18:2ω6 ct	12	0.20	0.22	0.07	0.14	0.33
C 18:2ω6 tc	12	0.16	0.17	0.06	0.09	0.28
C 18:2ω6 cc	12	21.00	22.34	6.32	14.20	34.5
C 20:0	12	0.04	0.06	0.03	0.03	0.13
C 18:3ω6	12	0.30	0.33	0.11	0.17	0.49
C 20:1ω9	12	0.14	0.15	0.03	0.10	0.19
C 18:3ω3	12	0.53	0.89	0.99	0.36	3.30
c9, t11-CLA	12	0.19	0.20	0.02	0.16	0.25
t10, c12-CLA	12	<0.03	<0.03			
C 18:4ω3	4	0.13	0.13	0.04	0.09	0.17
C 20:2ω6	12	0.19	0.19	0.02	0.17	0.24
C 22:0	11	0.13	0.12	0.05	0.04	0.19
C 20:3ω6	12	1.30	1.27	0.20	0.86	1.50
C 20:4ω6	12	4.30	4.11	0.95	2.50	5.30
C 20:5ω3 (EPA)	12	0.75	1.12	0.72	0.29	2.50
C 22:4ω6	12	0.19	0.17	0.08	0.04	0.28
C 22:5ω3 (DPA)	12	0.56	0.60	0.19	0.38	0.96
C 22:6ω3 (DHA)	12	1.40	1.93	1.19	0.83	4.30
Total fatty acids ( μg/g)	12	2571	2778	573	2111	3918

*) The daily supplementation was 4 × 500 mg CLA Capsules with a total amount of about 700 mg c9, t11-CLA and 700 mg t10, c12-CLA.

These results suggest that the main source of CLA in the diet is milk and dairy products. Also, this finding is in agreement with studies, which revealed that dairy products, especially cheeses due to their high fat content, are rich CLA sources [6,7,23]. On the other hand, 32 of the 77 samples of the second group presented amounts equal to or higher than the lower limit of the third group (supplementation group). The others show lower levels than the lower CLA level of the supplementation group. These data indicate that the supplementation can increase the CLA content of the human blood plasma and is therefore an alternative source of CLA for people who suffer from dairy allergies or do not consume dairy products for other reasons. These results comply with previous ones presented by Mougios *et al *[21].

It was additionally of interest to find out, if the CLA supplementation has an influence on the composition of the total amount of saturated and unsaturated fatty acids and on the ratio of ω-6- to ω-3-fatty acids in the blood plasma.

The corresponded comparison data of the three groups were calculated from the tables 1, 2 and 3 and are expressed as % the total fatty acids (Table 4). The CLA supplementation shows obvious a significantly effect on the total saturated fatty acids and the ω-6-fatty acids. CLA reduces the total saturated fatty acids as well as the total ω-6-fatty acids and especially the arachidonic acid. This corresponds to a reduction of the ratio of ω-6-fatty acids and arachidonic acid to ω-3-fatty acids. CLA appears to react as antagonist against ω-6-fatty acids and arachidonic acid. The last is of importance, because a low ratio of ω-6- to ω-3-fatty acids is a valuable determinant of health [22,24]. These effects of CLA on saturated acids and ω-6-fatty acids have to be confirm through further studies with a higher number of test persons.

**Table 4:** Comparison data of the total saturated and unsaturated blood Plasma fatty acid composition (% of total fatty acids) of groups 1, 2 and 3

**SATURATED AND UNSATURATED**		**GROUP 1**	**GROUP 2**	**GROUP 3**
**FATTY ACIDS (%)**		**AVERAGE % OF TOTAL FATTY ACIDS**		
Total saturated fatty acids		38.57	36.47	34.11
Total monounsaturated fatty acids		27.08	26.44	26.10
Total polyunsaturated fatty acids,		34.34	37.08	33.71
of them	ω-6-fatty acids*,	32.06	30.22	26.45
of them	arachidonic acid	6.83	5.41	4.11
	ω-3-fatty acids*	4.10	3.22	3.65
Ratio of ω-6 to ω-ω-fatty acids*		7.80	9.38	7.24
Ratio of Arachidonic acid to EPA+DPA+DHA		1.66	1.68	1.12

*) The essential ω-6-fatty acids linoleic acid and arachidonic acid and the essential eicosanoids ω-3-fatty acids EPA, DPA and DHA have been only considered.

The present study could be characterized as a first step in the direction of establishing a normal CLA content of human blood plasma. Based on these results, it could be suggested that the lower limit of the blood plasma CLA content is approximately 0.1% of total fatty acids. Thus, individuals who present rumenic acid levels within the range of the first group, namely up to 0,09% of total fatty acids, should consider increasing their CLA uptake through dairy products and/or supplements and could serve as ideal participants in future CLA supplementation studies.

## Competing interests

The authors declare that they have no competing interests.

## Authors' contributions

SNZ conceived, designed and coordinated the study. KL carried out the experiments. AS performed the statistical analysis. All authors read and approved the final manuscript.
